# Factors influencing the length of hospital stay of people experiencing homelessness

**DOI:** 10.3389/fpubh.2025.1545377

**Published:** 2025-03-11

**Authors:** Renate Karpenko, Sonia Lech, Liane Schenk, Daniel Schindel

**Affiliations:** ^1^Charité – Universitätsmedizin Berlin, Corporate Member of Freie Universität Berlin and Humboldt-Universität zu Berlin, Institute of Medical Sociology and Rehabilitation Science, Berlin, Germany; ^2^Charité – Universitätsmedizin Berlin, Corporate Member of Freie Universität Berlin and Humboldt-Universität zu Berlin, Department of Psychiatry and Neurosciences, Berlin, Germany

**Keywords:** ill-housed persons, hospitalization, length of stay, comorbidity, ICD-10, sex characteristics, health inequities

## Abstract

**Introduction:**

People experiencing homelessness (PEH) are affected by poor mental and physical health. Crucial healthcare remains inaccessible. In urgent need, people seek assistance in hospitals. The length of stay (LOS) can be used as an indicator of quality in inpatient healthcare. This study aimed to reveal factors influencing the LOS of PEH.

**Methods:**

A retrospective secondary data analysis of hospital discharge letters was conducted. Descriptive analyses were used to examine sociodemographics and the LOS in relation to individual disease groups according to the ICD-10. Disease burden was evaluated using a modified Elixhauser Comorbidity Score (ECS). Analyses were conducted separately by sex. Multiple linear regression was used to identify factors influencing the LOS.

**Results:**

The analysis included 807 hospital discharge letters from 521 PEH. The majority of letters were from men (89.2%). Both groups differed significantly in terms of age, with more women under the age of 30 years (27.1% versus 10.3%, *p* < 0.001). The total median LOS was 7 days with no sex difference (IQR women: 3.5–11.5, IQR men: 3–12, *p* = 0.837). Women had the longest median LOS for infectious diseases, skin diseases, and mental disorders. Men had the longest median LOS for infectious diseases, musculoskeletal diseases, and respiratory diseases. The median ECS was zero for both (*p* = 0.548). Significant factors influencing the LOS included mental disorders (*β*: 0.327, B: 0.788, CI(B): 0.465–1.110, *p* < 0.001), infectious diseases (*β*: 0.240, B: 0.869, CI(B): 0.504–1.234, *p* < 0.001), and homelessness duration (*β*: 0.213, B: 0.059, CI(B): 0.031–0.086, *p* < 0.001).

**Conclusion:**

Gender had no significant effect on the LOS. The significant demographic factor was the duration of homelessness, indicating that the health status of PEH deteriorates and access to healthcare decreases over time. Medical factors had a strong influence on the LOS of PEH. In highly prevalent disease categories, PEH have long hospital stays. A relevant factor for the LOS of PEH is their health status. Improving care structures has the potential to improve the LOS. Early integration of healthcare and social work can ensure a safe discharge and influence the LOS. The development of adequate aftercare services for PEH is necessary.

## 1 Introduction

Homelessness is a social and health policy challenge (1–3). The precarious living conditions of individuals experiencing homelessness make them particularly susceptible to poor physical and mental health (3–7). The access to healthcare for people experiencing homelessness (PEH) is limited (3, 8–12). Lacking regular outpatient care, most of the PEH use medical support in hospitals, if an urgent need is apparent (3, 8, 9, 13–16). Hospitals and emergency departments, along with low-threshold outpatient care, therefore form part of the care structure for PEH (8, 15, 16). One way to measure the quality of healthcare in hospitals is the length of stay (LOS) (17, 18). In accordance with the World Health Organization, the LOS also serves as an indicator of healthcare facility efficacy (19). The LOS affects health outcomes such as readmission rates and mortality of patients (17, 20). It has been demonstrated that an increased length of hospital stay is associated with a reduced probability of survival (20). In a detailed review, Buttigieg et al. identified different main categories affecting the LOS of non-homeless individuals (21). Patient characteristics influencing the LOS include demographic factors and medical factors. The clinical caregivers’ characteristics that were mentioned were multidisciplinarity and discharge planning (21). Research suggests that PEH remain in the hospital for a longer period of time than people not experiencing homelessness and homelessness is associated with an increased LOS (22–26). Still, only limited studies have investigated potential factors that influence the LOS of PEH. To date, factors that have been described for PEH include schizophrenia or bipolar disorder, high outpatient consultations, the severity of mental disorders, injuries, being older than 65 years, and being Hispanic (versus white) (22, 27–29). According to Buttigieg et al., there may be more relevant influence factors on the LOS of PEH (21).

In people not experiencing homelessness, demographic factors include being older, unemployed, unmarried, and female gender as associated with prolonged LOS (21, 30–33). As far as gender is concerned, research indicates that women experience poorer health than men (34, 35). According to the World Health Organization, gender is one of the core social determinants in population health and health inequities (36, 37). Although the mortality of women is generally lower than men’s (38), women report their health status worse than men (39). In particular, in the group of young women between 18 and 29 years, the difference is significant (39). Women with lower socioeconomic status report even worse health (39). The reasons therefore are multitude and intertwined. First, a greater sensitivity with regard to body and health and a greater willingness to accept medical help are discussed (40, 41). Second, the female gender itself has a negative influence on health outcomes as proposed by Hilton et al. (42). As a result of the enduring legacy of gender inequality in a patriarchal social system, the health consequences are severe for women (37, 43). According to Heise et al. different pathways to health are gendered. Women and men still differ in terms of employment and engagement in the care economy (i.e., care work for children and other family members), resulting in different exposures and possibilities of prioritizing your own health needs (34, 43). They also exhibit different health behaviors, which is reflected in a different vulnerability to diseases. Women also face a gender-biased health system and gender-biased health research (43). Women’s health-related complaints are often dismissed or downplayed due to stereotypes (e.g., being emotional), leading to their physical symptoms being overlooked. Today’s research, especially in the field of cardiovascular care, proves that in high-income countries women often receive lower quality care than men (43). In developing countries due to limited resources such as income and legal rights, also access to healthcare is impacted by gender (37, 43). Although health disparities in people not experiencing homelessness are described, so far, the knowledge about gender differences in the health of PEH is scarce.

In people not experiencing homelessness, women have higher rates of outpatient and inpatient healthcare utilization (44). Studies also suggest gender differences in hospital usage of PEH. Women experiencing homelessness have higher rates in recent usage of acute healthcare and recent emergency department visits (45–48). Beijer et al. proposed that women experiencing homelessness face a higher risk of hospitalization for blood diseases (particularly anemia), infections (excluding HIV/AIDS), diseases of the genitourinary system, skin conditions, and neoplasms compared to men experiencing homelessness. The findings of Beijer et al. suggest that the greatest risk of hospitalization in general is observed among young women experiencing homelessness (aged 18–36 years) (49). In contrast, men experiencing homelessness have higher rates of injury and poisoning, diseases of the circulatory system (particularly ischemic heart disease), diseases of the digestive system (particularly diseases of the liver and pancreas), diseases of the respiratory system, and ear diseases (49). The current state of research is inconsistent and notably scarce regarding gender differences in the duration of hospitalization. As stated by Iwundu et al., women experiencing homelessness had a risk of more than double that of men experiencing homelessness of staying overnight in the hospital (47). Others proposed the LOS be extended for men experiencing homelessness (50). High rates of chronic medical conditions are noticed in women experiencing homelessness (45, 51, 52). Only a few studies have investigated whether women experiencing homelessness differ in any aspect of chronic diseases compared to their male counterparts. There are hints that women experiencing homelessness may be affected by a higher disease burden: they have a 2.5-fold increased risk of reporting fair or poor health status and have significantly more health problems (53); 33 to 40% of women experiencing homelessness report fair or poor health, and 55% have a current physical limitation (51, 53). One of the predictors to report poor health was being women (53). Being a woman is associated with a moderate-to-high physical symptom burden (54). This disease burden of women experiencing homelessness could reflect on the LOS and lead to gender differences in hospitalization.

Important medical factors include the severity of diseases and the comorbidity burden (21). The lack of health insurance, experiences of discrimination, problems with transportation or financial resources, and competing basic human needs make it particularly challenging for PEH to access (regular) healthcare (9, 10, 55–57). If medical issues get to the point of being undeniable, emergency departments are used because they cannot be rejected there (8, 15, 16). As a result, PEH presenting themselves at hospitals tend to exhibit severe manifestations of physical and mental diseases (3). Hwang et al. have previously proposed that the severity of mental disorders is relevant in the context of prolonged LOS of PEH (29).

In addition to the severity of the disease itself, comorbidities represent another potential patient characteristic of PEH. The prevalence of multimorbidity in PEH is higher than in people not experiencing homelessness (58). The current research indicates that between 20 and 60% of PEH are concurrently affected by two or more chronic medical conditions (59–64). With 76.2%, the prevalence of mental disorders is especially high among PEH (7). In addition to substance misuse, every eighth homeless individual has depression or a disorder of the schizophrenia spectrum (7). Psychiatric diseases in general are proven to lead to prolonged LOS of people not experiencing homelessness (65–67). In PEH, Russolillo et al. found that schizophrenia and bipolar disorder were independent predictors of the LOS (27).

PEH also have a high prevalence of somatic diseases (3, 5). Schindel et al. found that in Germany infectious diseases, cardiovascular diseases, musculoskeletal diseases, and respiratory diseases are common (5). Skin diseases and injuries were also frequently referenced, particularly among individuals sleeping rough (5). Due to insufficient opportunities for personal hygiene and lack of accommodation, communicable diseases such as hepatitis A, pediculosis, and scabies are highly prevalent (68). Up to 56% of PEH are affected by scabies, whereas up to 22% are affected by lice (69). Missing adequate accommodation elevates the risks of skin diseases as well. Periods of lying on hard surfaces result in pressure injuries. The inability to lie down causes lower extremity stasis dermatitis (68). Ultimately, both can result in the development of ulcers. In people not experiencing homelessness, chronic ulcers lead to prolonged LOS (70). Research on the LOS in relation to different diseases is very limited for PEH and people not experiencing homelessness. Hypothesizing a high comorbidity burden of PEH may reflect on the LOS.

The potential characteristics of clinical caregivers mentioned include multidisciplinary expertise and discharge planning. Patients who require more intensive social work intervention during hospitalization due to social problems, including issues related to their home situation, tend to have longer LOS (71). Social work is an essential part of PEH’s healthcare, but multidisciplinarity may affect the LOS. The discharge planning process is complex for PEH because medical facilities for post-discharge treatments are rare. Hwang et al. proposed that the lack of sufficient aftercare programs is a reason for delayed discharge among PEH (29). PEH frequently encounter delayed discharges and a deficiency of essential post-discharge care structures (72). With regard to the provision of healthcare, it is worth noting that the structure of healthcare differs worldwide. This could also have an impact on the LOS. In insurance-based systems (e.g., Germany and the USA), economic pressures could lead to premature discharges. Publicly funded healthcare systems (e.g., Sweden and Canada) may have longer inpatient stays. However, the issue of limited post-discharge care structures applies to both systems (29, 72).

Concluding the aim of the following study is to describe the medical care of PEH in hospitals, particularly with regard to the LOS. The analysis includes sociodemographic and calculations of the LOS for different ICD-10 disease categories. The special focuses are gender differences and disease burden. Leading research questions were as follows: How long do PEH stay in hospital? How high is the disease burden of PEH? What factors influence the LOS of PEH? What are the gender differences?

## 2 Materials and methods

### 2.1 Study design, data basis, and data entry

This is a retrospective secondary data analysis of patient records data of the Health Center for the Homeless of the Jenny De la Torre Foundation in Berlin (Germany). Patients with at least one hospital discharge letter were included in the present study. Those hospital discharge letters were either brought to the health center by the patients themselves or sent directly to the center by the hospitals. The health center opened in 2006. The study comprises data from patient files from the opening of the health center until 2020 (the beginning of the study project). The health center is a low-threshold donation-financed care facility that offers a wide range of free support in the form of social, psychological, legal, and medical support. Furthermore, it provides PEH with food, clothing, a shower, and hairdressing. The social anamnesis was carried out by the team of the health center; the medical data were collected by the hospital staff. A total of 91% of all patient files of the health center were included in the study. A study protocol of the GIG study, whose data form the basis for the present study, was published prior to the beginning of the study (73).

#### 2.1.1 Sociodemographic factors that were analyzed included the following

Gender (female, male), age (divided into three age groups: ≤ 29 years, 30 to 44 years, ≥ 45 years), citizenship (divided into three groups: German citizenship, EU citizenship, non-EU citizenship), health insurance (yes, no), rough sleeping (i.e., sleeping in the streets and/or emergency shelters; yes, no), duration of school education (< 10 years of schooling, ≥ 10 years of schooling), school dropout (yes, no), at least 3 years of vocational training (yes, no), receiving social welfare (yes, no), marital status (single, married (together), married (separated), divorced, widowed), children (yes, no), contact with family or friends (yes, no), duration of homelessness (in years), and duration of unemployment (in years).

#### 2.1.2 Medical factors that were analyzed included the following

Number of hospital discharge letters, number of diagnoses per hospital discharge letter, length of stay (in days), length of stay (in days) according to ICD-10 group (using three-digit ICD-10-GM code (International Statistical Classification of Diseases and Related Health Problems, 10th Revision, German Modification)), comorbidity burden [calculated by number of Elixhauser comorbidities and Elixhauser Comorbidity Score by van Walraven et al. (74)].

The data entry was conducted between July 2020 and June 2022 in the health center. It was carried out by medical students on Internet-enabled laptops sponsored by Charité—University Medicine Berlin. As part of the treatment team, they screened the analog files for sociodemographic and medical factors and transferred them pseudonymously to a secured online database (REDCap, Research Electronic Data Capture Software). The pseudonyms were deleted afterward, so the analysis was carried out anonymously.

### 2.2 Ethic vote

This study uses the data from the GIG Study. The ethics commission of the Charité—University Medicine Berlin approved the GIG project (including the data protection concept), (EA1/058/20). It has been registered in the German Register of Clinical Studies (DRKS ID of the study: DRKS00021172).

### 2.3 Data analysis

First, a descriptive evaluation was carried out with regard to sociodemographic data by gender. For nominal and ordinal scaled data, the analysis was carried out using absolute and relative frequencies. Medians and interquartile ranges were calculated for metric variables. The medical data were analyzed in relation to ICD-10 main diagnosis groups stratified by gender. The LOS was evaluated by ICD-10 group and gender. The calculation of the LOS was performed for patients who were admitted to the hospital for at least 24 h (i.e., inpatient). The LOS was presented as the median and interquartile range because the data were not normally distributed. Supplementary Table 1 provides information on the LOS calculated for all patients (including patients with emergency department visits only). Comorbidities were assessed according to the definition of comorbidities by Elixhauser et al. (75). ICD-10 coding algorithms of Quan et al. were used to detect comorbidities (76). Some ICD-10 codes were not recorded in their entirety; therefore, all the ICD-10 codes needed to be shortened to an ICD-10 three-digit code. The Elixhauser Comorbidity Score (ECS) was calculated using the weighted sum score by van Walraven et al. (74). The usage of the ICD-10-three-digit code led to a modification of the originally proposed sum score. Of the originally proposed 21 comorbidities, 20 were considered because differentiation of blood loss anemia and deficiency anemia was not possible with the ICD-10 three-digit code. If one code was applied to two potential weights, the higher weight was used. For detailed information on modification of the weighted sum score (see Supplementary Table 2). Comorbidity classes were built in analogy to van Walraven et al. (74). The ECS ranges between −19 and + 89. Weighted sum scores and the comorbidity classes as proposed by van Walraven are non-linear associated with the health outcome as (74).

The dataset did not provide any information on diagnoses present on admission. Since a present on admission indicator was missing, all diseases of the discharge letters were taken into the calculation of the sum score. To identify significant group differences, the chi-square test, Fisher’s exact test, *t*-test, and Mann–Whitney *U*-test were used. Significant correlations were tested with Pearson correlation for metric variables, point-biserial correlation for dichotomous nominal variables, and ANOVA for nominal variables with more than two outcomes. A multiple linear regression was performed to predict the LOS, including covariates. Since the distribution was extremely right-skewed, a logarithmic transformation of the LOS variable was performed to reduce the asymmetry. Following the logarithmic transformation of the variable, all independent factors exhibiting a statistically significant correlation with the dependent variable were incorporated into the model. The analyses were carried out with SPSS 29.0. The selected significance level was *α* = 0.05.

## 3 Results

### 3.1 Description of the hospital sample

#### 3.1.1 Comparison with a total sample

A total of 3,338 patient files were initially reviewed, of which 19 were excluded due to unknown or diverse gender. The subsample (consisting of 521 patients with a hospital discharge letter) was comparable in sociodemographic characteristics to the total sample of 3,319 patients from the health center, including those without a discharge letter. Women were younger in both samples than men (total sample: women 33.3%, men 19.1% ≤ 29 years; subsample: women 27.1%, men 10.3% ≤ 29 years), in both samples, the majority of PEH identified as German nationals, regardless of gender (total sample: women 69.1%, men 69.7%; subsample: women 63.9%, men 61.5%), and in both samples, women were more likely to have health insurance than men (total sample: women 39.0%, men 33.4%; subsample: women 39.1%, men 27.0%). For further information on the total sample see Supplementary Table 3.

#### 3.1.2 Number of letters

Of the total collective of 3,319 PEH, 521 (15.7%) had at least one hospital discharge letter, of which 72 (13.8%) were women and 449 (86.2%) were men. These 521 PEH form the basis of the analyses. Of the PEH who had at least one letter in their patient file, 77.9% (*n* = 406) had exactly one letter, 12.9% (*n* = 67) had two letters, and 9.2% (*n* = 48) had at least three letters in their file, with the maximum being 32 letters for one patient. From 807 hospital discharge letters, 720 (89.2%) were from men experiencing homelessness and 87 (10.8%) from women experiencing homelessness. For further information on the number of letters, see Supplementary Table 4.

#### 3.1.3 Demographic factors

Women differed significantly from men in terms of age profile (*p* < 0.001). Nearly one in three women (27.1%) treated in the hospital were younger than 30 years, while half of the men (51.5%) were at least 45 years old. The two groups exhibited comparable citizenship profiles with the majority being German. A significantly greater proportion of women than men had health insurance at the time of admission (women: 39.1%, men: 27.0%, *p* = 0.045). Women experiencing homelessness were significantly less likely to sleep rough (women: 50%, men: 68.4%, *p* = 0.003). The distribution of schooling levels was equal in the group comparison. Women were significantly less likely to have a vocational school qualification (women: 55%, men: 71.4%, *p* = 0.044). The majority of both received social welfare and both groups did not differ significantly in the duration of unemployment or the duration of homelessness. Marital status differed in the group comparison (*p* = 0.035). Women were significantly more likely to have at least one child (women: 63.6%, men: 45.6%, *p* = 0.032). A greater proportion of both groups reported no contact with family or friends (women: 54.8%, men: 59.5%, *p* = 0.691) (Table 1).

**Table 1:** Sociodemographic of hospital sample.

	Women (*n* = 72)		Men (*n* = 449)		*p*
	*n*	%	*n*	%	*p*
Age category					<0.001
≤ 29 years	19	27.1	46	10.3	
30–44 years	21	30.0	171	38.3	
≥ 45 years	30	42.9	230	51.5	
(missing)	2	-	2	-	
Citizenship					0.869
German	39	63.9	228	61.5	
EU	16	26.2	109	29.4	
Non-EU	6	9.8	34	9.2	
(missing)	11	-	78	-	
Health insurance					0.045
Yes	27	39.1	114	27.0	
No	42	60.9	308	73.0	
(missing)	3	-	27	-	
Sleeping rough					0.003
Yes	36	50.0	307	68.4	
No	36	50.0	142	31.6	
(missing)	0	-	0	-	
School education					1.00
< 10 years	17	44.7	92	44.9	
≥ 10 years	21	55.3	113	55.1	
(missing)	34	-	244	-	
School dropout					0.751
Yes	4	12.1	17	9.6	
No	29	87.9	160	90.4	
(missing)	39	-	272	-	
Vocational training					0.044
Yes	22	55.0	157	71.4	
No	18	45.0	63	28.6	
(missing)	32	-	229	-	
Social welfare					0.873
Yes	19	55.9	90	52.3	
No	9	26.5	54	31.4	
Other	6	17.6	28	16.3	
(missing)	38	-	277	-	
Marital status					0.035
Single	19	46.3	153	67.1	
Married (together)	1	2.4	4	1.8	
Married (separated)	1	2.4	12	5.3	
Divorced	16	39.0	49	21.5	
Widowed	4	9.8	10	4.4	
(missing)	31	-	221	-	
Children					0.032
Yes	28	63.6	103	45.6	
No	16	36.4	123	54.4	
(missing)	28	-	223	-	
Social contact*					0.691
Yes	14	45.2	62	40.5	
No	17	54.8	91	59.5	
(missing)	41	-	296	-	
	Median	IQR	Median	IQR	
Duration of homelessness (in years)	3.0	2.0–5.0	5.0	2.0–8.0	0.102
(missing)	57	-	337	-	
Duration of unemployment (in years)	11.5	6.25–19.25	9.0	4.0–14.0	0.094
(missing)	52	-	302	-	

*Contact with family members or friends.

### 3.2 Number of diagnoses and total inpatient LOS

The majority of discharge letters contained exactly one diagnosis (women: 43.7%, men: 34.3%). The distribution of the number of diagnoses was similar. The median number of diagnoses per letter was two in both groups; the interquartile range was greater for men. Women and men differed significantly in the median number of diagnoses (*p* = 0.007). Men were more likely than women to have at least five diagnoses (women: 14.9%, men: 26.3%, *p* = 0.025) (Table 2).

**Table 2:** Number of diagnoses and total inpatient LOS.

	Women (*n* = 87)		Men (*n* = 720)		*p*
	*n*	%	*n*	%	
Number of diagnoses					0.073
1	38	43.7	247	34.3	
2	20	23.0	120	16.7	
3	9	10.3	102	14.2	
4	7	8.0	62	8.6	
≥ 5	13	14.9	189	26.3	
At least 5 diagnoses (yes)	13	14.9	189	26.3	0.025
	Median	IQR	Median	IQR	
Number of diagnoses	2	1–3	2	1–5	0.007
	WEH (*n* = 37)		MEH (*n* = 401)		
	Median	IQR	Median	IQR	
Total LOS (in days)	7	3.5–11.5	7	3–12	0.837

The total median LOS for inpatient women experiencing homelessness and men experiencing homelessness was 7 days (IQR women: 3.5–11.5, IQR men: 3–12), with no significant intergroup differences (*p* = 0.837) (Table 2).

### 3.3 Inpatient LOS according to the ICD-10

Women experiencing homelessness had the longest median LOS in discharge letters that included at least one diagnosis of the category of certain infectious and parasitic diseases (A00-B99). The median LOS was 23.5 days (IQR: 8–39) in this group. The median LOS for women diagnosed with at least one disease of the group diseases of the skin and subcutaneous tissue (L00-L99) was 2 weeks (IQR: 2.75–9.25). The third longest median LOS was found in hospital discharge letters indicating at least one mental or behavioral disorder (F00-F99), with a median of 9.5 days (IQR: 4.25–19.25) (Table 3).

**Table 3:** Inpatient LOS according to the ICD-10.

	WEH (*n* = 37)			MEH (*n* = 401)			
ICD-10 Code	*n*	Median	IQR	*n*	Median	IQR	*p*
A00-B99 Certain infectious and parasitic diseases	4	23.5	8–39	72	10.5	5–20	0.224
E00-E90 Endocrine, nutritional and metabolic diseases	10	5.5	2.75–9.25	86	8	4–13.25	0.203
F00-F99 Mental and behavioral disorders	20	9.5	4.25–19.25	258	8	4–14	0.497
I00-I99 Diseases of the circulatory system	11	8	4–17	141	8	5–14	0.807
J00-J99 Diseases of the respiratory system	5	7	5.5–10	70	8.5	4–15.25	0.761
K00-K93 Diseases of the digestive system	3	7	7-	119	8	4–14	0.533
L00-L99 Diseases of the skin and subcutaneous system	4	14	2.75–20.75	68	8	5–16.75	0.727
M00-M99 Diseases of the musculoskeletal system and connective tissue	5	5	3–16.5	26	9.5	4–21	0.538
S00-T98 Injury, poisoning, and certain other consequences of external causes	8	4.5	1.75–10	114	5	3–10	0.652

In parallel with their female counterparts, a review of the median LOS for men experiencing homelessness revealed that the longest median LOS was observed among those with at least one disease classified under the category of certain infectious and parasitic diseases (A00-B99). The median LOS for this group was 10.5 days (IQR: 5–20). The next group comprised hospital discharge letters of men with at least one musculoskeletal system disease (M00-M99), with a median hospital stay of almost 10 days. The median LOS for this group was 9.5 days (IQR: 4–21). The third longest median LOS was observed in hospital discharge letters of men with at least one diagnosis of respiratory disease (J00-J99). The median LOS was 8.5 days (IQR: 4–15.25) in this group (Table 3).

There was no significant group difference comparing the LOS according to the ICD-10.

### 3.4 Comorbidity burden of PEH

The median ECS was zero for women experiencing homelessness and men experiencing homelessness, with no significant group difference (*p* = 0.353). The distribution of the ECS classes was similar (*p* = 0.548). In both groups, nearly every tenth patient showed an ECS of lower than zero (women: 9.2%, men: 9.9%). The majority in both groups showed an ECS of zero (women: 59.8%, men: 57.1%) (Table 4).

**Table 4:** ECS classes, total median ECS, and number of Elixhauser comorbidities.

	Women (*n* = 87)		Men (*n* = 720)		*p*
	*n*	%	*n*	%	
ECS Class					0.353
<0	8	9.2	71	9.9	
0	52	59.8	411	57.1	
1–5	13	14.9	82	11.4	
6–13	12	13.8	98	13.6	
>14	2	2.3	58	8.1	
	Median	IQR	Median	IQR	
ECS	0	0–4	0	0–4	0.548
No. of Elixhauser comorbidities	1	0–2	1	0–2	0.380

### 3.5 Influence factors on the LOS

The LOS was estimated using multiple linear regression models including covariates. The final model explained 41.3% of the variance in the LOS (*R*^2^ = 0.413, adj. *R*^2^ = 0.393). The model was statistically significant (*F* (8, 240) = 21.08, *p* < 0.001). Significant influence factors on the LOS were having at least one mental or behavioral disorder (F00-F99), having at least one certain infectious or parasitic disease (A00-B99), the duration of homelessness, the number of Elixhauser comorbidities, and having at least one disease of the digestive system (K00-K93) (Table 5).

**Table 5:** Influence factors on the LOS.

Factor	B	SE (B)	*β*	*t*	*p*	Confidence interval for B	
						Lower limit	Upper limit
(Constant)	0.164	0.302		0.543	0.588	−0.431	0.758
Age category	−0.036	0.107	−0.018	−0.337	0.737	−0.248	0.175
Gender	0.162	0.247	0.033	0.656	0.513	−0.325	0.650
At least one mental or behavioral disorder (F00-F99)	0.788	0.164	0.327	4.809	**0.000**	**0.465**	**1.110**
At least one certain infectious or parasitic disease (A00-B99)	0.869	0.185	0.240	4.695	**0.000**	**0.504**	**1.234**
Duration of homelessness	0.059	0.014	0.213	4.221	**0.000**	**0.031**	**0.086**
Number of Elixhauser comorbidities	0.140	0.050	0.201	2.803	**0.005**	**0.042**	**0.239**
At least one disease of the digestive system (K00-K93)	0.383	0.158	0.137	2.427	**0.016**	**0.072**	**0.693**

B, Unstandardized regression coefficient; SE (B), standard error (of B); *β*, standardized regression coefficient; *t*, *t* statistics, *p*, *p*-value. Bold values indicates significant influence factors.

## 4 Discussion

This study aimed to describe the medical care of PEH in hospitals, particularly with regard to the LOS. Significant factors that influenced the LOS were medical factors and the duration of homelessness. The analyses of the demographic patient characteristics such as age and gender showed no significant influence on the LOS. In particular, gender turned out to be no factor influencing the LOS. Women and men experiencing homelessness had the same median LOS of 7 days in total. Stahl-Toyota et al. found a median LOS of 6 days for their sample of over 28,000 German inpatients with a mean age of 64 years (65). Big samples of people not experiencing homelessness from the USA, Canada, Switzerland, and Israel exhibit a median LOS of 4 to 5 days (74, 77, 78). No significant difference in the LOS in different diagnostic categories between genders could be detected. Still, women exhibited more than 3 weeks for infectious diseases and 2 weeks for skin diseases the longest median LOS of the total sample. This finding may be relevant as women experiencing homelessness have a higher risk of hospitalization in these disease categories than men experiencing homelessness (49). Although women were slightly younger than men, the disease burden measured by ECS was comparable in both groups. This could indicate a worse health status for women experiencing homelessness as proposed by previous studies (51, 53, 54). Nonetheless, gender turned out to be no significant influencing factor on the LOS in the regression model, although for people not experiencing homelessness previously described (30, 32, 33). In addition to the influence of differing health behaviors and experiences of gender-related stereotyping in healthcare and research, social variables such as education, income, and occupational or employment status have been identified as potential sources of health disparities (34, 43, 44). In our sample, with the exception of vocational training, women and men experiencing homelessness exhibited no difference in regard to schooling levels, income (i.e., receiving social welfare), or duration of unemployment. Marital status and being a caregiver influence health outcomes as well. Although women were significantly more likely to have children, both reported to have no social contact at all, including any family member. The marital status differed in both groups; nonetheless, the proportion of being married and together was similar in both groups, and the majority in both groups reported being single. Small differences in sociodemographics may not have a great influence on the LOS, given the same comorbidity burden between women and men experiencing homelessness. Given the significantly higher number of younger women in the group, it can be assumed that their health is worse despite the identical median LOS in total. This effect could have been more accurately represented by a larger sample size. Further studies are necessary to confirm this assumption.

As a novel finding, the duration of homelessness was the only demographic factor with significant influence on the LOS of PEH. An association between homelessness and the LOS was postulated previously (26). Women and men had a similar duration of homelessness. It seems reasonable to assume that a lengthy period of homelessness results in severe somatic and psychiatric diseases, which, in turn, may affect the LOS. In conclusion, it can be assumed that the similarities in terms of sociodemographics, but, especially in terms of morbidity, resulted in a comparable LOS between women and men in this sample.

Significant influence factors of the LOS were medical patient characteristics. Four different disease categories and the number of Elixhauser comorbidities were associated with the LOS. Women and men experiencing homelessness exhibited the longest durations of hospitalization if being diagnosed with mental disorders, infectious diseases, skin diseases, musculoskeletal or respiratory diseases. Those diseases are highly prevalent in PEH (3, 5, 7). The absence of regular medical care can lead to a higher disease burden of PEH on admission (3). Mental disorders had the biggest influence on the LOS in our sample. That aligns with the conclusions of limited preceding studies that psychiatric diseases and their severity influence the LOS of PEH (27, 29). In addition to mental and behavioral disorders, infectious and parasitic diseases, diseases of the skin, and diseases of the digestive system showed significant influence on the LOS. PEH are at major risk for infectious diseases and skin diseases as a result of their harsh living (68). In the context of diseases of the skin, stasis dermatitis and chronic ulcers represent a significant concern among PEH (50, 68). In people not experiencing homelessness, chronic ulcers elevate the risk of infections and complications and lead to a prolonged LOS (70). This finding seems applicable to PEH. To our knowledge, it is a novel finding that infectious diseases and digestive diseases significantly influence the LOS of PEH. Infectious diseases are a more common reason for hospitalization of women experiencing homelessness; digestive diseases are a more common reason for hospitalization of men experiencing homelessness.

To quantify the disease burden, a modified Elixhauser Comorbidity Score by van Walraven was used (74). The ECS, in its original, is a valuable tool for measuring comorbidity burden in people not experiencing homelessness (74, 77, 79, 80). The median ECS in this sample was zero, with no gender differences. An ECS of zero indicates that the comorbidity burden is comparable to that of people not experiencing homelessness or even lower. The median ECS varies depending on the examined population, ranging from zero to 18; a median ECS of five is common (66, 74, 78, 81–83). The original sample of van Walraven et al. showed an ECS of zero with an interquartile range of zero to eight (74). A potential explanation for a median ECS of zero may be that compared with people not experiencing homelessness, the percentage of psychiatric diagnoses was high in our sample. Particularly, the percentage of drug abuse, which is assigned to the lowest possible value of −7 in the ECS, was strikingly high. Every tenth individual was diagnosed with a drug abuse diagnosis. This is more than two times the proportion compared with the original sample of van Walraven et al. (74). This accounting could result in a higher negative score in total. The distortion due to psychiatric illnesses could result in a lower total ECS, indicating a high mental comorbidity burden. Because of the high prevalence of mental diseases, the ECS as a pure sum score proposed by van Walraven seems to be only suitable to a limited extent for PEH. The ECS also showed a lower correlation with the outcome variable LOS than the number of diagnoses. The pure number of Elixhauser comorbidities, on the other hand, was shown to be a greater influencing factor of the LOS of PEH. Therefore, the number of Elixhauser comorbidities could represent the morbidity burden more adequately. With a higher number of Elixhauser comorbidities, the LOS increases. This suggests that the number of comorbidities influences the LOS of PEH significantly.

As potential characteristics of clinical caregivers influencing the LOS, PEH require more intensive social work during their hospitalization. In-hospital assessment and support from social workers are associated with increased LOS (71). An earlier involvement of social work and a multidisciplinary approach could therefore influence the LOS of PEH. The discharge planning process is more complex for PEH than for people not experiencing homelessness. Hwang et al. proposed that one reason for delayed discharge among PEH is the lack of sufficient aftercare programs (29). Particularly for skin diseases, lack of post-discharge help may increase the LOS because diseases of the skin necessitate daily care (70). After the discharge, lack of accommodation and medical supplies make the daily care of skin diseases challenging for PEH (50). In Berlin, there is a single medical facility for PEH that provides necessary medical and nursing care following hospital treatment (84). Previous studies have demonstrated the efficacy of aftercare structures in improving the healthcare situation of PEH (72, 85, 86). As a consequence, an expansion of those medical aftercare programs could influence the LOS of PEH.

The only demographic factor influencing LOS was the duration of homelessness. In particular, there was no significant influence of gender on the LOS. Although the proportion of men over 30 years was significantly greater, they showed the same median LOS of 7 days and the same ECS of zero as women.

### 4.1 Limitations

The results are limited by the very nature of the data itself. Only one healthcare center was analyzed, which elevates the risk of selection bias and potentially reduces the generalizability to all PEH. Furthermore, it should be noted that not every individual undergoing treatment at the health center brought a letter to the center, or indeed, that the letter was sent to the center at all. This does not indicate that the individual was not admitted to the hospital during their treatment at the health center. In addition, a lot of missing data made the analyses difficult. Particularly regarding sociodemographic factors, the data basis was limited, and the sample of women was small so that no further differentiating analyses could be conducted. As only ICD-10 three-digit codes were used, another limitation is that the accurate distinction of comorbidities was not possible. Consequently, the originally used ECS of van Walraven had to be modified, and the ECS was calculated differently. This could make direct comparisons less precise. The data did not provide any information on if the disease was already present on admission. A present on admission indicator could help differentiate between comorbidities and acute complications that arise during the hospitalization period. Missing information on diagnoses being present on admission, all diagnoses mentioned in the letters were included in the analysis. It could result in an overestimation of the disease burden. Ultimately, the LOS was calculated based on ICD-10 categories if at least one disease was mentioned in the letter. Focusing on one disease category could neglect the intertwined effects of multiple diseases.

## 5 Conclusion

The results highlight the importance of accessible healthcare to reduce morbidity in the group of PEH, especially in the group of younger women experiencing homelessness. Further investigation is required to ascertain whether women, particularly those under the age of 30, are at an elevated risk for poor health outcomes. A multidisciplinary approach is essential for the health of PEH. Social workers should be involved at an early stage to ensure a safe discharge. Adequate aftercare structures could reduce barriers to discharge. As longer periods of homelessness are associated with poorer health, the most effective solution to improve the healthcare of PEH may be the reduction of homelessness duration itself.

## Data Availability

The data analyzed in this study is subject to the following licenses/restrictions: the data analyzed in this study consists of patient records (analog files and hospital discharge letters) stored at a low-threshold facility for individuals experiencing homelessness. A digital version of selected data is securely stored in the “REDCap” database. Access to this data is strictly limited to authorized staff due to privacy and security requirements. External requests for data access may be considered on a case-by-case basis but are subject to applicable data protection laws and institutional policies. Requests to access these datasets should be directed to renate.karpenko@charite.de.
