# Supplementary material for: Factors influencing the length of hospital stay of people experiencing homelessness
Source: Front Public Health. 2025 Mar 11;13:1545377. doi: 10.3389/fpubh.2025.1545377 (PMC11933102; doi:10.3389/fpubh.2025.1545377)
Supplement: Supplementary file 1 [file Data_Sheet_1.docx]

Supplementary Material

# Supplementary Figures and Tables

## Supplementary Tables

**Supplementary Table 1.** The LOS including PEH receiving emergency department treatment only.

|  | **Women (n=86)** | | | **Men (n=705)** | | |  |
| --- | --- | --- | --- | --- | --- | --- | --- |
| **ICD-10 Code** | **n** | **Median** | **IQR** | **n** | **Median** | **IQR** | **p** |
| **A00-B99  Certain infectious and parasitic diseases** | 8 | 2.5 | 0-26.75 | 88 | 8 | 1.25-18.5 | .445 |
| **E00-E90  Endocrine, nutritional and metabolic diseases** | 15 | 3 | 0-7 | 98 | 7 | 3-11.25 | .025 |
| **F00-F99  Mental and behavioral disorders** | 26 | 6.5 | 0.75-12.5 | 317 | 6 | 1-11.5 | .915 |
| **I00-I99  Diseases of the circulatory system** | 16 | 4 | 0-10 | 171 | 6 | 2-11 | .284 |
| **J00-J99  Diseases of the respiratory system** | 6 | 6.5 | 3.75-10 | 85 | 6 | 1-12.5 | .848 |
| **K00-K93  Diseases of the digestive system** | 6 | 3.5 | 0-15.75 | 136 | 7 | 2-12 | .257 |
| **L00-L99  Diseases of the skin and subcutaneous system** | 7 | 1 | 0-20 | 99 | 6 | 0-13 | .754 |
| **M00-M99  Diseases of the musculoskeletal system and connective tissue** | 8 | 3 | 0-10.25 | 47 | 2 | 0-10 | .977 |
| **S00-T98  Injury, poisoning and certain other consequences of external causes** | 32 | 0 | 0-0.75 | 260 | 0 | 0-5 | .04 |

**Supplementary Table 2.** Modification of the ECS according to van Walraven et al. (2009).

| **Disease Category** | **ICD-10 Code** | **3-digit ICD-10 Code** | **Included ICD-10 Code** | **Value** |
| --- | --- | --- | --- | --- |
| **Congestive heart failure** | I09.9, I11.0, I13.0, I13.2, I25.5, I42.0, I42.5– I42.9, I43.x, I50.x, P29.0 | I09, I11, I13, I25, I42, I43, I50, P29 | I09, I11, I13, I25, I42, I43, I50, P29 | 7 |
| **Cardiac arrhythmias** | I44.1–I44.3, I45.6, I45.9, I47.x–I49.x, R00.0, R00.1, R00.8, T82.1, Z45.0, Z95.0 | I44, I45, I47, I48, I49, R00, T82, Z45, Z95 | I44, I45, I47, I48, I49, R00, T82, Z45, Z95 | 5 |
| **Valvular disease** | A52.0, I05.x–I08.x, I09.1, I09.8, I34.x–I39.x, Q23.0–Q23.3, Z95.2– Z95.4 | A52, I05, I06, I07, I08, I09, I34, I35, I36, I37, I38, I39, Q23, Z95 | A52, I05, I06, I07, I08, I34, I35, I36, I37, I38, I39, Q23 | -1 |
| **Pulmonary circulation disorders** | I26.x, I27.x, I28.0, I28.8, I28.9 | I26, I27, I28 | I26, I27, I28 | 4 |
| **Peripheral vascular disorders** | I70.x, I71.x, I73.1, I73.8, I73.9, I77.1, I79.0, I79.2, K55.1, K55.8, K55.9, Z95.8, Z95.9 | I70, I71, I73, I77, I79, K55, Z95 | I70, I71, I73, I77, I79, K55 | 2 |
| **Hypertension, uncomplicated** | I10.x | I10 | Item excluded from the analysis because its value (=0). | 0 |
| **Hypertension, complicated** | I11.x–I13.x, I15.x | I11, I12, I13, I15 | Item excluded from the analysis because its value (=0). | 0 |
| **Paralysis** | G04.1, G11.4, G80.1, G80.2, G81.x, G82.x, G83.0–G83.4, G83.9 | G04, G11, G80, G81, G82, G83 | G04, G11, G80, G81, G82, G83 | 7 |
| **Other neurological disorders** | G10.x–G13.x, G20.x– G22.x, G25.4, G25.5, G31.2, G31.8, G31.9, G32.x, G35.x–G37.x, G40.x, G41.x, G93.1, G93.4, R47.0, R56.x | G10, G11, G12, G13, G20, G21, G22, G25, G31, G32, G35, G36, G37, G40, G41, G93, R47, R56 | G10, G12, G13, G20, G21, G22, G25, G31, G32, G35, G36, G37, G40, G41, G93, R47, R56 | 6 |
| **Chronic pulmonary disease** | I27.8, I27.9, J40.x–J47.x, J60.x–J67.x, J68.4, J70.1, J70.3 | I27, J40, J41, J42, J43, J44, J45, J46, J47, J60, J61, J62, J63, J64, J65, J66, J67, J68, J70, | J40, J41, J42, J43, J44, J45, J46, J47, J60, J61, J62, J63, J64, J65, J66, J67, J68, J70, | 3 |
| **Diabetes, uncomplicated** | E10.0, E10.1, E10.9, E11.0, E11.1, E11.9, E12.0, E12.1, E12.9, E13.0, E13.1, E13.9, E14.0, E14.1, E14.9 | E10, E11, E12, E13, E14 | Item excluded from the analysis because its value (=0). | 0 |
| **Diabetes, complicated** | E10.2–E10.8, E11.2–E11.8, E12.2– E12.8, E13.2–E13.8, E14.2–E14.8 | E10, E11, E12, E13, E14 | Item excluded from the analysis because its value (=0). | 0 |
| **Hypothyroidism** | E00.x–E03.x, E89.0 | E00, E01, E02, E03, E89 | Item excluded from the analysis because its value (=0). | 0 |
| **Renal failure** | I12.0, I13.1, N18.x, N19.x, N25.0, Z49.0– Z49.2, Z94.0, Z99.2 | I12, I13, N18, N19, N25, Z49, Z94, Z99 | I12, N18, N19, N25, Z49, Z99 | 5 |
| **Liver disease** | B18.x, I85.x, I86.4, I98.2, K70.x, K71.1, K71.3– K71.5, K71.7, K72.x– K74.x, K76.0, K76.2– K76.9, Z94.4 | B18, I85, I86, I98, K70, K71, K72, K73, K74, K76, Z94 | B18, I85, I86, I98, K70, K71, K72, K73, K74, K76, Z94 | 11 |
| **Peptic ulcer disease excluding bleeding** | K25.7, K25.9, K26.7, K26.9, K27.7, K27.9, K28.7, K28.9 | K25, K26, K27, K28 | Item excluded from the analysis because its value (=0). | 0 |
| **AIDS/HIV** | B20.x–B22.x, B24.x | B20, B21, B22, B24 | Item excluded from the analysis because its value (=0). | 0 |
| **Lymphoma** | C81.x–C85.x, C88.x, C96.x, C90.0, C90.2 | C81, C82, C83, C84, C85, C88, C96, C90 | C81, C82, C83, C84, C85, C88, C96, C90 | 9 |
| **Metastatic cancer** | C77.x–C80.x | C77, C78, C79, C80 | C77, C78, C79, C80 | 12 |
| **Solid tumor without metastasis** | C00.x–C26.x, C30.x–C34.x, C37.x–C41.x, C43.x, C45.x–C58.x, C60.x–C76.x, C97.x | C00, C01, C02, C03, C04, C05, C06, C07, C08, C09, C10, C11, C12, C13, C14, C15, C16, C17, C18, C19, C20, C21, C22, C23, C24, C25, C26, C30, C31, C32, C33, C34, C37, C38, C39, C40, C41, C43, C45, C46, C47, C48, C49, C50, C51, C52, C53, C54, C55, C56, C57, C58, C60, C61, C62, C63, C64, C65, C66, C67, C68, C69, C70, C71, C72, C73, C74, C75, C76, C97 | C00, C01, C02, C03, C04, C05, C06, C07, C08, C09, C10, C11, C12, C13, C14, C15, C16, C17, C18, C19, C20, C21, C22, C23, C24, C25, C26, C30, C31, C32, C33, C34, C37, C38, C39, C40, C41, C43, C45, C46, C47, C48, C49, C50, C51, C52, C53, C54, C55, C56, C57, C58, C60, C61, C62, C63, C64, C65, C66, C67, C68, C69, C70, C71, C72, C73, C74, C75, C76, C97 | 4 |
| **Rheumatoid arthritis/ collagen vascular diseases** | L94.0, L94.1, L94.3, M05.x, M06.x, M08.x, M12.0, M12.3, M30.x, M31.0– M31.3, M32.x–M35.x, M45.x, M46.1, M46.8, M46.9 | L94, M05, M06, M08, M12, M30, M31, M32, M33, M34, M35, M45, M46 | Item excluded from the analysis because its value (=0). | 0 |
| **Coagulopathy** | D65–D68.x, D69.1, D69.3– D69.6 | D65, D66, D67, D68, D69 | D65, D66, D67, D68, D69 | 3 |
| **Obesity** | E66.x | E66 | E66 | -4 |
| **Weight loss** | E40.x–E46.x, R63.4, R64 | E40, E41, E42, E43, E44, E45, E46, R63, R64 | E40, E41, E42, E43, E44, E45, E46, R63, R64 | 6 |
| **Fluid and electrolyte disorders** | E22.2, E86.x, E87.x | E22, E86, E87 | E22, E86, E87 | 5 |
| **Blood loss anemia** | D50.0 | D50 | Summarized with deficiency anemia on the basis of the same ICD-10 code (=D50) | - |
| **Deficiency anemia** | D50.8, D50.9, D51.x–D53.x | D50, D51, D52, D53 | Summarized with blood loss anemia on the basis of the same ICD-10 code (=D50) | -2 |
| **Alcohol abuse** | F10, E52, G62.1, I42.6, K29.2, K70.0, K70.3, K70.9, T51.x, Z50.2, Z71.4, Z72.1 | F10, E52, G62, I42, K29, K70, T51, Z50, Z71, Z72 | Item excluded from the analysis because its value (=0). | 0 |
| **Drug abuse** | F11.x–F16.x, F18.x, F19.x, Z71.5, Z72.2 | F11, F12, F13, F14, F15, F16, F18, F19, Z71, Z72 | F11, F12, F13, F14, F15, F16, F18, F19, Z71, Z72 | -7 |
| **Psychosis** | F20.x, F22.x–F25.x, F28.x, F29.x, F30.2, F31.2, F31.5 | F20, F22, F23, F24, F25, F28, F29, F30, F31 | Item excluded from the analysis because its value (=0). | 0 |
| **Depression** | F20.4, F31.3–F31.5, F32.x, F33.x, F34.1, F41.2, F43.2 | F20, F31, F32, F33, F34, F41, F43 | F20, F31, F32, F33, F34, F41, F43 | -3 |

**Supplementary Table 3.** Description of the total sample (n=3319).

|  | **Women (n=716)** | | **Men (n=2603)** | | **p** |
| --- | --- | --- | --- | --- | --- |
|  | **n** | **%** | **n** | **%** | **p** |
| **Age category** |  |  |  |  | <.001 |
| ≤ 29 years | 232 | 33.3 | 493 | 19.1 |  |
| 30-44 years | 232 | 33.3 | 965 | 37.4 |  |
| ≥ 45 years | 232 | 33.3 | 1124 | 43.5 |  |
| (missing) | 20 | - | 21 | - |  |
| **Citizenship** |  |  |  |  | .948 |
| German | 404 | 69.1 | 1492 | 69.7 |  |
| EU | 125 | 21.4 | 445 | 20.8 |  |
| Non-EU | 56 | 9.6 | 203 | 9.5 |  |
| (missing) | 131 | - | 463 | - |  |
| **Health insurance** |  |  |  |  | .008 |
| Yes | 254 | 39.0 | 804 | 33.4 |  |
| No | 398 | 61.0 | 1606 | 66.6 |  |
| (missing) | 64 | - | 193 | - |  |
| **Sleeping rough** |  |  |  |  | .187 |
| Yes | 443 | 61.9 | 1681 | 64.6 |  |
| No | 273 | 38.1 | 922 | 35.4 |  |
| (missing) | 0 | - | 0 | - |  |
| **School education** |  |  |  |  | .249 |
| < 10 years | 133 | 37.2 | 546 | 40.6 |  |
| ≥ 10 years | 225 | 62.8 | 798 | 59.4 |  |
| (missing) | 358 | - | 1259 | - |  |
| **School drop out** |  |  |  |  | .508 |
| Yes | 25 | 7.8 | 109 | 9.1 |  |
| No | 296 | 92.2 | 1093 | 90.9 |  |
| (missing) | 395 | - | 1401 | - |  |
| **Vocational training** |  |  |  |  | <.001 |
| Yes | 193 | 51.5 | 977 | 67.5 |  |
| No | 182 | 48.5 | 477 | 32.8 |  |
| (missing) | 341 | - | 1149 | - |  |
| **Social welfare** |  |  |  |  | .007 |
| Yes | 178 | 57.1 | 619 | 51.2 |  |
| No | 61 | 19.6 | 342 | 28.3 |  |
| Other | 73 | 23.4 | 247 | 20.4 |  |
| (missing) | 404 | - | 1395 | - |  |
| **Marital status** |  |  |  |  | .003 |
| Single | 259 | 64.9 | 1063 | 72.2 |  |
| Married (together) | 16 | 4.0 | 34 | 2.3 |  |
| Married (separated) | 33 | 8.3 | 69 | 4.7 |  |
| Divorced | 79 | 19.8 | 282 | 19.2 |  |
| Widowed | 12 | 3.0 | 24 | 1.6 |  |
| (missing) | 317 | - | 1131 | - | .072 |
| **Children** |  |  |  |  |  |
| Yes | 187 | 44.6 | 597 | 39.7 |  |
| No | 232 | 55.4 | 907 | 60.3 |  |
| (missing) | 297 | - | 1100 | - | .066 |
| **Social contact** |  |  |  |  |  |
| Yes | 137 | 49.8 | 460 | 43.6 |  |
| No | 138 | 50.2 | 596 | 56.4 |  |
| (missing) | 441 | - | 1547 | - |  |
|  | **Median** | **IQR** | **Median** | **IQR** |  |
| **Duration of homelessness**  **(in years)** | 2 | 1-4 | 3 | 2-7 | <.001 |
| (missing) | 570 | - | 1984 | - |  |
| **Duration of unemployment**  **(in years)** | 5 | 2-10 | 6 | 3-11 | .055 |
| (missing) | 562 | - | 1781 | - |  |

**Supplementary Table 4.** Number of letters of PEH.

|  | **Women (n=72)** | | **Men (n=449)** | |
| --- | --- | --- | --- | --- |
| **Number of letters** | **n** | **%** | **n** | **%** |
| **1** | 62 | 86.1 | 344 | 76.6 |
| **2** | 6 | 8.3 | 61 | 13.6 |
| **3** | 3 | 4.2 | 20 | 4.5 |
| **4** | 1 | 1.4 | 9 | 2.0 |
| **5** | - | - | 2 | .4 |
| **6** | - | - | 3 | .7 |
| **7** | - | - | 4 | .9 |
| **8** | - | - | 1 | .2 |
| **9** | - | - | 1 | .2 |
| **13** | - | - | 1 | .2 |
| **20** | - | - | 2 | .4 |
| **32** | - | - | 1 | .2 |
